# The Effect of Musical Environments on Designers’ Attention: Persistent Music Listening Interferes with Attention

**DOI:** 10.3390/bs14030216

**Published:** 2024-03-06

**Authors:** Shulan Yu, Xinran Chen

**Affiliations:** 1 Department of Information and Interaction Design, College of Furnishings and Industrial Design, Nanjing Forestry University, Nanjing 210037, China; 2 Department of Information and Interaction Design, College of Art and Design, Nanjing Forestry University, Nanjing 210037, China; chenxinran@njfu.edu.cn

**Keywords:** designers, Attention Network Test, creativity, music

## Abstract

Research indicates that music can influence human cognitive functions. Diverse musical settings can affect alertness, orientation, and executive control of attention in various populations. Exploring the relationship between designers with highly creative thinking and music environments can provide new research perspectives for the cognitive field. A total of 94 students, consisting of 61 design majors and 33 non-design majors, completed the Attention Network Test (ANT) on a computer under three test environments: cheerful music, melancholic music, and silence. The study results indicated that the alerting network effect between the design professional group and the control group was marginally significant. However, there were no significant differences between the groups in the orienting subsystem and the executive control subsystem. Within the design professional group, the attentional network data indicated that participants showed improved performance in alerting and orienting attention in a music-free environment compared to cheerful and melancholic music environments (*pa* = 0.028, *po* = 0.008). Nevertheless, executive control attention did not show significant differences across the music environments. In conclusion, existing research confirms that designers are more susceptible to distraction from external stimuli; thus, music-free environments assist them in concentrating.

## 1. Introduction

### 1.1. Attention and Attention Network Testing

Attention permeates nearly all facets of human behavior. Thus, it has a prominent position in cognitive psychology. Attention aids in prioritizing information processing and in generating or retaining information without external input, which is crucial for processing working memory (WM) [1]. Working memory is strongly linked to attention, and workers’ sustained attention and the nature of their activity impact their working memory [2]. This leads to sustained attention, which refers to the ability to actively analyze recurrent stimuli without being distracted by other stimuli that might cause habituation [3,4]. This ability is crucial and frequently utilized for advanced cognitive functioning in many everyday life and professional tasks. Attention can be categorized into three types: focused attention, selective attention, and broad attention. Focused attention involves identifying and responding to task-related information. Selective attention entails making positive choices and responding to task-relevant information while ignoring distracting external stimuli. Broad attention involves giving feedback and responding to multiple tasks simultaneously [5].

Posner and Petersen categorized attention into three subsystems: alerting, orienting, and executive control [6]. Fan et al. quantified three attention subsystems using the Attention Network Test (ANT) to assess alerting, orienting, and executive attention [7]. The alerting network can achieve and maintain readiness to respond to environmental signals [8]. The orienting network directs attention to specific stimuli in the environment to prioritize objectives [9], while executive control manages interference and resolves conflicts [10]. Meanwhile, multiple studies have demonstrated that the alerting network inhibits executive control, the orienting network enhances executive control, and the alerting network regulates orienting effects [11,12,13]. The test system is commonly utilized for attention assessment in adolescents, the elderly, and patients [7,14,15]. For example, Baijal et al. conducted a study where adolescents who had received centralized meditation training (CMT) for at least one year were asked to complete the ANT task. The study compared their results to those of a control group and found that CMT improved alerting and executive control subsystems but did not significantly enhance orienting [14]. Dovorany et al. conducted the ANT on cognitively healthy older individuals in various musical settings, revealing that both cheerful and melancholic music enhanced attention in older adults. Specifically, cheerful music improved alertness in the older age group, while melancholic music enhanced executive control [15].

Participants complete the Attention Network Test on a computer by performing a flanker task to determine if the central arrow points left or right. Their reaction times (RTs) are recorded to assess the three subsystems. The task can be classified into three types based on the direction of the arrows: congruent trials (all arrows point in the same direction), incongruent trials (center arrow points opposite to flanker arrows), and neutral trials (center arrow without flanker arrows). Prior to the initiation of the flanker task, participants receive several alert cues: directional cues indicating the flanker task’s location or no cues, requiring participants to determine the flanker task’s presentation location themselves [7]. McConnell and Shore analyzed how cueing and flanker conditions are interconnected in ANT experiments and discovered that the alerting, orienting, and executive control networks can collaborate to influence behavior, as indicated by the behavioral data collected from the ANT [16].

### 1.2. Creative Thinking

Designers are acknowledged for being an exceptionally creative cohort. Design is commonly acknowledged as a creative profession, with good designers being themselves creative people, so their work is frequently characterized as creative [17]. A designer’s role involves focusing on the form and function of the design goal. Form refers to creating unique and innovative objects, while function involves customizing the design to meet the user’s requirements. Designers must use adaptable thinking to balance various design criteria that impact originality and suitability [18,19]. Creativity is intricately linked to intellect, making its assessment thorough and intricate [20]. Questionnaire-based measures can be self-evaluated through personality inventories [21], thinking style inventories [22], and self-reported creative activities and accomplishments [23]. Task-based measures can be assessed by experts through divergent thinking (DT) tasks [24], artistic and real-life creative tasks [25], insight tasks [26], and so on. Many scholars in the field of creative thinking utilize a mix of scales and activities to evaluate the creative talents of the target group for a comprehensive assessment [18,27].

Creativity is intricately linked to attention. Existing research indicates a strong correlation between creativity and attention. Neurophysiological studies show that highly creative individuals tend to filter out fewer external stimuli, a phenomenon known as attentional leakage. This leakage allows individuals to focus on out-of-focus ideas and incorporate them into their current thought process, which can enhance creative thinking [28]. Several studies indicate that having a wide focus enhances creativity [29,30,31]. Creative high achievers spontaneously direct their attention to “seemingly irrelevant” sensory information in the environment within 50 ms of a sensory stimulus [32]. Unlike highly creative achievers, individuals with elevated levels of divergent thinking excel at screening out sensory stimuli considered “irrelevant” [28]. Zabelina et al.’s research confirms that divergent thinkers have a more flexible attention span, though tests measuring divergent thinking mostly emphasize concentration and inhibition rather than divergent thinking skills [33].

### 1.3. The Mozart Effect and the Arousal Hypothesis

The Mozart effect indicates that fast-tempo music in rhythmic and major modes can enhance arousal and well-being, but slow music in minor modes might reduce arousal and evoke feelings of melancholy [34,35]. Thompson et al. conducted a test to assess the effects of cheerful music (Mozart’s sonata) and melancholic music (Albinoni adagio) on participants. The test results indicated that individuals who listened to cheerful music outperformed those who listened to melancholic music. Furthermore, participants who listened to cheerful music indicated that the music heightened their arousal and enjoyment [34]. Similarly, Xing et al. demonstrated experimentally that rhythmic structure plays a crucial role in the Mozart effect by studying the impact of Mozart K. 488 and its retrograde version on humans and rats. They found that the similarity between the music’s melodic patterns and physiological cycles is what allows Mozart K. 448 to enhance mood and arousal across different species [36]. However, there is no unanimous agreement among academics on the Mozart effect. Within three years following the proposal, other scholars duplicated the experiment but did not discover the Mozart effect [37,38,39]. Several studies have shown that the Mozart effect is ineffective in children [40] and musicians [41] and does not improve epileptic patients [42] or enhance adults’ situational memory [43]. Thus, the existence and reliability of the Mozart effect remain a subject of serious controversy.

In contrast to the Mozart effect, Husain contended that music’s cognitive enhancements might be attributed to the “arousal of emotions” hypothesis [44]. Arousal and mood are distinct emotional reactions, but they exhibit some degree of correlation. Valence refers to the intensity of emotions, encompassing both positive emotions (joy, happiness, fondness, etc.) and negative emotions (sadness, anger, frustration, etc.) [45]. Arousal is the intensity of physiological or psychological activation [46]. In Russell’s (1980) Circumplex Model of Affect, affect is represented by two orthogonal dimensions: arousal and valence [44]. Overall, the “arousal” hypothesis proposes that music affects cognitive functioning through emotional responses rather than directly improving cognitive emotional responses [45]. This contradicts the “Mozart effect” theory, which proposes that music enhances cognitive abilities in individuals. Nantes and Schellenberg’s tests, which reproduced the “Mozart effect” by exposing subjects to Mozart’s sonata plus a narrative plot, support the “arousal” hypothesis. The results indicated that individuals performed better on space mission tasks when exposed to the audio of their preferred stimulus. For instance, those who like Mozart’s music demonstrated improved performance on the spatial tests after listening to the Mozart sonata, and vice versa. Mozart’s music had no bearing on the participants’ cognitive improvement; rather, it was the particular aural stimuli they subjectively chose that played a significant role in increasing their arousal levels and emotional responses [47]. Xia et al.’s experiments corroborated this hypothesis [48].

### 1.4. Purpose and Hypothesis

This study aimed to examine how various musical settings (cheerful and fast-paced music, melancholic and slow-paced music, and no music) impact designers while they complete an Attention Network Test. The existing literature [14,15,48] shows that no comparable experiments have been conducted on the population of designers, a group known for their high level of creativity. Therefore, considering the findings, the following two hypotheses were formulated:

**Hypothesis H1:**  *Cheerful music has a positive effect on the alerting network and executive control in the design professional group.*

**Hypothesis H2:**  *A decrease in environmental stimuli is positively correlated with an increase in orienting network metrics in the design professional group.*

All the above hypotheses were measured using RT differences in the three subsystems of the attention network.

## 2. Materials and Methods

### 2.1. Participants

The sample size was calculated using G*Power with the effect size *f* = 0.25, *α* = 0.05, and 1 − *β* = 0.8, and the required sample size was calculated to be 86 people. A total of 94 individuals were recruited to participate in the experiment, as per the calculated sample size. These 94 participants were recruited from fourth-year undergraduate to third-year graduate students at the researchers’ university. There were 61 design majors (33 males and 28 females) and 33 engineering students in the control group (21 males and 12 females), as shown in Table 1. The participants were Chinese native speakers with no medical record of mental illness and normal or corrected eyesight. The students in the design professional group and the control group were randomly assigned to the cheerful music group, melancholic music group, and music-free group by a random drawing process. Participants were instructed to refrain from performing vigorous activity and consuming caffeinated beverages for 2–3 h prior to the test and to ensure they had 7–8 h of sufficient sleep the night before. Upon completion of the experiment, each participant received a payment of RMB 10. The study received approval from the Ethics Committee of the college, and all participants signed a written informed consent form before the experiment.

### 2.2. Methods

#### 2.2.1. Procedure

**Figure 1:**
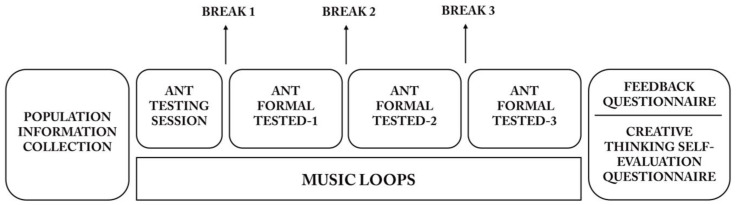
Experimental design: Participants complete a demographic information form and then proceed to a quiet, empty space to fill out the ANT. The ANT consists of a pretest and a formal test, with participants being given a break at the conclusion of each test. Background music loops in both the cheerful and melancholic music groups, with participants in each group filling out a questionnaire following the experiment.

The subjects in each group (professional and control) were randomly assigned (by lottery) to one of three experimental groups, two with music and one without music, with the design averaging 20 participants per group in the professional group and 11 per group in the control group. The introduction to the study included a description of the experiment, the experimental procedure, the pre-experiment precautions, and the informed consent form. The experiment was conducted in a quiet and unoccupied space. All subjects completed the ANT on an HP Pavilion Gaming Laptop 15-cxOxxx. Each subject completed the same experimental component (Figure 1). The task was programmed using E-Prime 2.0 (psychology lab software). The ANT is divided into four parts. The first part is a practice phase, designed to allow subjects to understand how the program works and what will be tested. The remaining three parts are formal tests, each lasting approximately 5 min, with a break provided after each section. The test procedure lasted 20–25 min.

#### 2.2.2. Musical Stimulus

Since the experimental space was quiet enough, the music was looped in the form of external playback using a Sony SRS-XB13 player. The player was placed diagonally in front of the computer, and the music was played at a sound level of 60 dB. The selection of cheerful music was Mozart’s *Sonata for Two Pianos in D major*, movement 1, K. 448, and the melancholic music was Albinoni’s *Adagio in G minor for strings and organ* [45]. The length of the cheerful music piece was 5:59 min, and the length of the melancholic music piece was 4:45 min. Each piece of music was played from the beginning of the subject’s ANT task and looped until the subject completed all tasks.

#### 2.2.3. Questionnaire

Prior to the test, the participants received an information collection form and an explanation of the experiment. Data were gathered on their name, age, gender, grade level, major, and musical training. At the end of the ANT, participants were required to assess their emotional state post-listening to the music on a scale of 0 (melancholic) to 100 (cheerful). They were also asked to indicate their familiarity with the musical piece by choosing from three options: A. Never heard of it, not familiar at all; B. Seem to have heard of it, somewhat familiar; C. Have heard of it, very familiar. Additionally, they provided information on their musical background, including vocal or instrumental training, and the duration of their training. Furthermore, the participants were given a Creative Thinking Self-Evaluation Questionnaire that had questions on creative feedback perception [49] and employed the Creative Personality Scale [21] (CPS).

Liu developed the Creative Feedback Perception Questionnaire [49], a self-assessment measure known for its excellent reliability and validity. The scale is structured into four factors: individual positive feedback, individual negative feedback, task positive feedback, and task negative feedback. Each factor consists of four questions. The scale can measure individual feedback on creativity. The CPS is a well-recognized and widely used instrument for measuring creative personality [50], comprising 18 positive and 12 negative adjectives. Participants receive 1 point for selecting a positive term and −1 point for selecting a negative term, resulting in scores that can range from −12 to 18 [21]. The scale has been used to measure the creativity of artists across several disciplines, as well as to evaluate the effectiveness of creativity training and the creativity levels of students [18].

The Creative Feedback Perception Questionnaire responds to external evaluations of an individual’s creativity, and the CPS targets creativity in personality. This study evaluates individuals’ levels of creativity in these two aspects.

#### 2.2.4. Attention Network Test

All subjects completed the improved version based on ANT 3.0.2002. The participants used a 17.3-inch laptop screen positioned 50–55 cm away to complete activities by following on-screen instructions using the laptop keyboard and left mouse button. Subjects were instructed to keep their attention on the center of the screen in order to perform a task involving a set of five arrows displayed horizontally. The task included a congruent condition where the central arrow matched the direction of the surrounding arrows, an incongruent condition where the central arrow pointed in the opposite direction, and a neutral condition where the central arrow was surrounded by straight lines with no arrows. One of four visual signals, denoted by an asterisk (“*”), was delivered before the flanker stimulus. (1) No cue: no “*” appeared after the gaze point; (2) Double cue: an “*” symbol appeared both above and below the gaze point; (3) Central cue: an “*” symbol appeared at the gaze point location; (4) Spatial cue: individual “*” symbols appeared above or below the gaze point, and a cue for the flanker task followed the cue symbols in all cases, establishing the spatial cues as valid.

**Figure 2:**
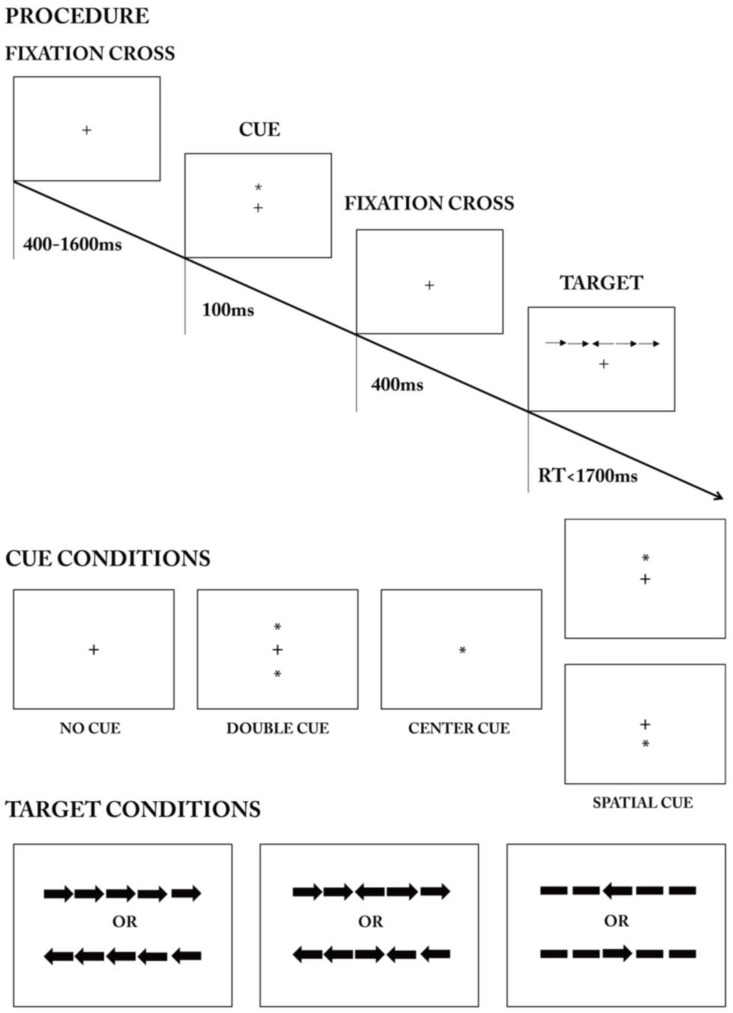
Attention network experimental procedure, cue conditions, and target conditions in the experiments.

Each sub-trial commenced with a gaze point symbol “+” displayed for 400–1600 ms, succeeded by a cue shown for 100 ms, then a central gaze point displayed for 400 ms. Following this, a flanking stimulus appeared above or below the central gaze point, prompting the subjects to promptly assess the direction of the central arrow. When the middle arrow pointed left, the subject punched the “F” key, and when it pointed right, the subject pressed the “J” key. The participant had to respond within 1700 ms, and the stimulus vanished as soon as the keyboard key was pressed (Figure 2). The technique was conducted once, with a set total duration of 4000 ms for each experiment. The software logged the subject’s reaction time and accuracy for every attempt. The practice phase included 24 trials, and each set of formal experiments had 72 trials, totaling 240 trials for the whole experiment. Each cue was shown 54 times, and each surrounding stimulus was presented 72 times.

## 3. Results

### 3.1. Analysis of Background Music Perception

The data were analyzed using SPSS version 25 (IBM (Armonk, NY, USA), 2017). The participants initially decided on the level of musical instruction. According to the findings, 80.34% of the participants lacked any musical training. Of those who did, 75.08% had received training within one year. An independent-sample *t*-test was utilized to compare the mood scores of all participants following exposure to cheerful and melancholic background music. There was a notable contrast in the participants’ evaluations of the emotional aspects of the two musical compositions. The cheerful music received higher ratings (*M* = 84.69, *SD* = 8.793) compared to the melancholic music (*M* = 42.58, *SD* = 12.374) on the music rating scale, with a significant difference (*t* = 15.608, *p* < 0.001). The data on participants’ acquaintance with the two pieces of background music were not normally distributed; hence, a nonparametric test was used for analysis. Table 2 shows that there was no statistically significant difference in participants’ familiarity with the two pieces of music (*U* = 445.000, *n*1 = 32, *n*2 = 31, *p* = 0.394, two-sided). Frequency analysis was utilized to determine the participants’ familiarity with the background music as a percentage. In total, 42 individuals (66.67%) were unfamiliar with the topic, 19 (30.16%) were moderately familiar, and 2 (3.17%) were highly familiar. Overall, 96.83% of participants selected options A and B together.

### 3.2. Creativity Scale

This study aimed to determine if there was a disparity in creativity levels between the professional and control groups. A univariate, two-level independent-sample *t*-test was performed on the groups using the scores from the Creative Feedback Perception Scale as the dependent variable. The professional group achieved considerably higher scores than the non-professional group (*t* = 2.188, *p* = 0.031; refer to Table 3 for scale scores by group). A *t*-test was performed on the CPS data, revealing no significant difference between the groups (*t* = 0.838, *p* = 0.404).

### 3.3. Changes in Attention in Different Musical Environments

Reaction times (RT; *ms*) were determined using valid data. As per the ANT principle developed by Fan et al., alerting attention was determined by subtracting the reaction time of no cue from the reaction time of double cue. No cue indicates more distracted attention, while a double cue enables the subject to achieve and sustain a state of alertness beforehand. A comparison of the two can demonstrate the subject’s capacity to regulate alertness, with a higher score suggesting a more robust alerting ability. The orienting network was derived by calculating the difference between the response time of the central cue and the response time of the spatial cue. Both cues provide temporal information, but the spatial cue is more successful in allowing individuals to anticipate the location of the flanker stimulus. Subtracting the two values can show the subject’s capacity to regulate orienting, with a higher result indicating a more robust orienting ability. The executive control subsystem was derived by subtracting the direction-inconsistent flanker task from the congruent flanker task. In the incongruent flanker task, where the center arrow conflicts with the surrounding arrows, subjects experience more interference and need to inhibit it using executive control. Therefore, completing the incongruent flanker task takes longer than the congruent flanker task. A smaller subtraction value indicates stronger executive control ability. The outcomes of the three subtractions between pairs are known as subsystem differences, and this analytical approach has been commonly utilized in many ANT investigations.

Table 4 displays the average differences between subsystems and their standard deviations for the design specialization group and the control group in various musical settings. The link between the ANT subsystems, disciplinary specialization, and musical environment was examined using a 2 × 3 repeated-measures analysis of variance (RMANOVA). Each analysis focused on the dependent variable (subsystem differences in alerting, orienting, and executive control RT), the specialty independent variable (design specialty group and control group), and the music environment independent variable (cheerful music, melancholic music, and music-free environment).

The study initially compared the attentional network performance of two groups (the professional design group and the control group) using a two-level independent-sample *t*-test to determine statistical significance. The alertness, orienting, and executive control subsystems were considered dependent variables. The independent-sample *t*-test results for the alerting subsystem indicated marginally significant differences between groups (mean difference = 7.648, *t* = 1.932, *p* = 0.056, Cohen’s *d* = 0.728). The control group exhibited lower time difference values for alert responses (RT no cue–RT double cue) compared to the professional group in both the cheerful music environment and the music-free environment, implying that the control group’s alerting attention in the same musical environment was superior. However, the independent-sample *t*-tests showed no significant differences between groups in both the orienting subsystem (mean difference = 1.427, *t* = 0.307, *p* = 0.760) and the executive control subsystem (mean difference = 6.264, *t* = 0.882, *p* = 0.380). Therefore, the overall results indicate that the control group exhibited higher attention levels in the alerting subsystem compared to the design professional group.

**Figure 3:**
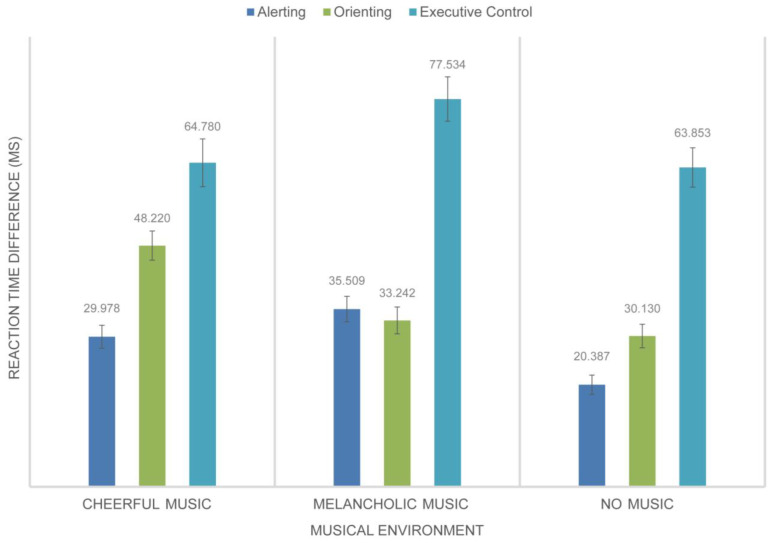
Difference in response time (*ms*) for attentional networks under different musical conditions in the design specialized group.

This study conducted a repeated-measures analysis of variance (RMANOVA) to further examine the impact of three different musical environments on the attentional network subsystems of design professionals. The three musical environments were cheerful music, melancholic music, and no music. The RT differences for alerting, orienting, and executive control within the attention network subsystems were the dependent variables in the analysis. The data results were shown in Table 5. The ANOVA findings for the alerting component showed a significant main impact of the music environment (*F* = 3.812, *p* = 0.028, *η*^2^ = 0.116). The participants in the design professional group showed considerably higher levels of alertness in the environment without music compared to the setting with cheerful music (*t* = 2.704, *p* = 0.008, Cohen’s *d* = 0.564). There was no significant difference between the cheerful music and melancholic music settings (*t* = 1.000, *p* = 0.321, Cohen’s *d* = 0.209) and between the music-free and melancholic music environments (*t* = 1.714, *p* = 0.092, Cohen’s *d* = 0.357), contradicting Hypothesis H1. The ANOVA findings indicated a significant main impact of the music environment on the orienting component (*F* = 4.301, *p* = 0.018, *η*^2^ = 0.129). The study revealed that participants’ orienting attention was significantly better in the melancholic music environment than in the cheerful music environment (*t* = 2.294, *p* = 0.025, Cohen’s *d* = 0.478) and also superior in the environment without music compared to the melancholic music environment (*t* = 2.737, *p* = 0.008, Cohen’s *d* = 0.571). This result confirmed Hypothesis H2. However, no significant difference was found between the absence of music and the cheerful music environment (*t* = 0.477, *p* = 0.635, Cohen’s *d* = 0.010). The ANOVA findings for the executive control component indicated a non-significant main impact of the music environment (*F* = 1.021, *p* = 0.367, *η*^2^ = 0.034), which contradicted Hypothesis H1, as seen in Figure 3.

## 4. Discussion

The present study aims to evaluate how different musical settings affect the attention of designers by determining which musical situations enhance their attentional subsystems. A total of 61 undergraduate and postgraduate design students from a university campus took an Attention Network Test, while 33 non-design majors in the same grade range served as the control group for the experiment. The between-group comparison showed that the alerting network was marginally significant (*p* = 0.056). The primary results for the attention network subsystem in the design professional group showed that the alerting subsystem was more effective when participants were in a music-free setting; therefore, Hypothesis H1 was not supported. The participants’ orienting subsystem measures were superior in the melancholic music environment compared to the cheerful music environment. However, this metric was better in the music-free setting than in the melancholic music environment, confirming Hypothesis H2. The various musical settings did not impact the participants’ executive control. The results are further elaborated on in the following sections.

### 4.1. Creativity Levels

The findings of the creativity scale indicated that there was no substantial disparity between the two groups in terms of creative personality, but there was a more pronounced distinction in terms of perceived creative feedback. This is believed to be connected to the grade of the subjects [51]. Simonton’s study revealed that college students reach the highest level of creativity during their junior year, followed by a subsequent decline [52]. This arises from the contrast between innovation and dogmatism. As college students progress through their academic years, their fields of study become more focused and tend to shift towards specialization, perhaps constraining the growth of creativity [53]. In contrast, leisure and positive emotions enhance creative performance [54], but creativity tends to decrease as students advance to their final year of college or graduate school due to rising scholastic and employment pressures. Since the subjects in this study ranged in grade level from the third year of college to the third year of graduate school, this may explain the absence of substantial differences in creative personalities.

Regarding the results of the Creative Feedback Perception Scale, we believe that design students are more likely to have encountered innovative work due to the prevalence of innovative design practice projects in the design program at the researchers’ university. Moreover, as the grade increases, the students’ design practice courses become richer. Consequently, individuals will receive more frequent and inventive feedback from external sources like professors and project team leaders, resulting in more positive personal and task feedback. Designers rely on external inventive input to evaluate the innovativeness and alignment with design criteria of their work [18], which enhances their sensitivity to it.

### 4.2. Comparison of Attention Networks in Two Groups

During an independent-sample *t*-test, the alerting network had borderline significance; however, the other two sub-networks did not exhibit significant differences, likely because of the smaller sample size in the control group. The between-group comparisons indicated that the designer group exhibited lower attentiveness. The designer group is acknowledged for their exceptional innovation. A designer’s main job at the beginning of a project is to generate more ideas and inspiration [55], as asserted by De Silva Garza [56]. Designers often need to have active and creative minds to explore more novel content, which then facilitates the development of a broad range of attention in this population [31]. Lunke and Meier examined the relationship between artistic creativity and attention and found that the visual arts were negatively correlated with attention through their own compilation of the ACDC questionnaire [57]. Tidikis et al. suggested that emotional valence and arousal are also relevant to attention and creativity [29], and their experimental results suggested that creative performance is determined by the type of task participants complete and that the effect of emotion on attention is determined by the combined impact of valence and arousal. This is consistent with the previous theory of the “arousal” hypothesis, in which music influences cognitive functioning and, thus, leads to changes in mood, further influencing attention [45,46]. This suggests that the music the design professionals in the experiment listened to did not significantly affect their mood. The background music selected did not improve the attention of the design professionals when faced with similar external conditions.

### 4.3. Analysis of Attention Network Subsystems within Design Specialty Groups

#### 4.3.1. Alerting

Prior studies have demonstrated that individuals engaging in an attentional network task while listening to cheerful music exhibit heightened alertness [15]. This study’s findings suggest that for the participants in the design professional group, no background music was more conducive to an increase in alerting attention, which is contrary to Hypothesis H1 suggesting that a cheerful music environment would increase attention in the design professional group. First, this is believed to be related to the form in which the music was played. Here, again, the definition of alerting attention needs to be clarified: achieving and maintaining a response to a signal or stimulus and being prepared for it, which responds to the fact that the alerting subsystem is used to maintain attention [8], i.e., the alerting network can be used as a measure of sustained attention. Previous studies have asked participants to listen to music prior to the ANT and to complete the testing task in a silent environment [15,58]. Participants in an intriguing study were divided into three groups to complete Chu’s attention exam: one group listened to music before the exam, another group completed the test without music, and the third group listened to music throughout the test. The experimental findings indicated that the first group outperformed the other groups in terms of mean score and total number of questions answered, while the third group fared the worst among the three groups in all instances [59]. Most participants in the design program (63.41% of the total) heard the background music for the first time, as indicated by their responses to the music familiarity questionnaire. This likely influenced the emotional arousal experienced by the participants. Linnell’s research demonstrated that participants’ acquaintance with the background music lessened their distraction levels and enhanced their RT [60]. Brown’s study found that unfamiliar musical environments would increase participants’ attention load, thus interfering with task performance [61]. The participants’ lack of familiarity with the music in the experiment, together with the genre of music, its presentation, and their personal preference, might affect their emotional arousal and intensity [59]. Finally, the results indicate that designers could be diverted by musical disruptions, aligning with the assumption that creative thinking is inversely connected to attention [31,57].

#### 4.3.2. Executive Control

This investigation failed to find relationships between various musical contexts and the executive control attention network. Previous research has indicated that listening to upbeat music can help improve executive control attention [34,44]. However, this experiment did not corroborate this finding. Executive control is used to monitor and resolve conflicts between competing mental processes [35], and its core function is to inhibit inappropriate task influences and screen for unwanted information [62]. Due to the singularity of the attentional network task operations [7] and the fact that the present experiment was set up with three tests, the participants became more familiar with the content of the tests over time. This could have caused a gradual reduction in their conflict inhibition level until it reached a point where they could effectively resolve a basic conflict and then stabilize. In other words, the common tasks and content did not require excessive executive control attention from the participants. Moreover, executive control attention is thought to involve cognitive and emotional self-regulation [63], which may be related to the participants’ familiarity with the music and the manner in which the music was played [62,63]. Consequently, the participants’ emotions did not impact their judgment of the flanker task direction. Furthermore, analyzing from the participants’ design professional background, previous studies have indicated that low executive control is linked to increased creativity levels. Experiments conducted by Radel et al. supported the idea that decreased inhibition could result in improved performance on open-ended creativity tasks [64]. The musical stimuli in this experiment may not have been strong enough to provide meaningful feedback to a group of designers with low inhibition, leading to no connection between the musical environment and the executive control network.

#### 4.3.3. Orienting

The experimental findings on orienting were in line with the notion that increasing external inputs reduces the activity of the orienting network. The external stimuli in the experimental setting were background music. The cheerful music was composed in rhythmic and major key patterns [34], and the melancholic music was composed with a slow rhythmic and minor key pattern [12]. The faster the tempo of the music, the more stimulating it is, i.e., cheerful music can provide more stimulation compared to melancholic music [64]. Thus, as the stimuli diminished (from cheerful to melancholic music) or disappeared (no music), the participants’ index of orienting attention improved. The notion of the orienting subsystem needs clarification: the capacity to choose particular information from various sensory stimuli [9]. Previous research has shown that orienting can be categorized as endogenous (top-down) or exogenous (bottom-up) and that both attentional orienting modalities can enhance cognitive performance, leading to increased neural activity in specific sensory systems [65]. Carmel noted that when individuals direct their attention to a specific type of target (e.g., a face) or a specific aspect of a target (e.g., a color), this leads to enhanced activity in specific neural areas for specific task-related computations [66]. This argument was supplemented by Cloutier et al., who mentioned that reducing the inhibitory effects of distracting stimuli is one way to increase neural activation [67], i.e., when an individual is focusing on a specific target, it reduces the inhibitory effects of other distracting stimuli on neural activity, allowing attention to be more focused on task-relevant information. This, then, further confirms the hypotheses of this paper for the orienting network. However, this result may be related to the form in which the music was played (it remained on a loop during the experiment) [58], which may have been a result of the distraction caused by the constant influence on the participants’ attention while carrying out the task. Therefore, in this context, background music becomes a negative interference factor. Additionally, we believe this is consistent with the theory that the familiarity of background music has a different effect on the emotional arousal of the participants [45], because emotional experience is closely related to cognitive processes. The results of the present experiment seem to be more consistent with the hypothesis of “arousal of emotions” from this point of view [44].

### 4.4. Shortcomings and Prospects

Overall, the present study indicates that while pleasant background music did not improve attention in designers when the music was unfamiliar, a music-free atmosphere enhanced their alertness and focus. This study is the first to investigate how various musical settings impact the attention of designers, although it has some deficiencies and constraints. The participants in the study attended the same college, but there were differences in their specific majors, so their inconsistencies in expertise may have affected the results of the experiment. However, it is worth considering that the attentional profile of practicing designers may differ from that of students due to their experience, which could be a potential area for future research. Furthermore, this study did not examine how various genders impact participants’ attention in diverse musical settings, therefore warranting further investigation. Moreover, restrictions were present in how the music was presented to the participants in this experiment, and the participants’ familiarity with the music was not adequately considered. Moreover, this study did not comprehensively consider the confounding factors of the selected subjects’ music training and music listening habits; those with more experience in music training have been shown to have stronger neural responses to music [68], and music listening habits affect the arousal of human emotions [69]. Finally, limitations existed in measuring the individuals’ creative levels in this study. Future studies might employ a more thorough assessment to study individual creativity, thereby enabling a better analysis of the relationships between creativity and attention, music, and specialization.

## Figures and Tables

**Table 1:** Demographic information on gender, grade level, and major for design majors (professional group *n* = 61) and non-design majors (control group *n* = 33).

Group	Gender		Grade		Major	
**Professional Group**	Male	33	Fourth-Year University Study	8	Applied design students	40
			First-Year Graduate Study	14		
	Female	28	Second-Year Graduate Study	4	Academic design students	21
			Third-Year Graduate Study	4		
**Control Subjects**	Male	21	Fourth-Year University Study	9	Applied engineering students	33
			First-Year Graduate Study	13		
	Female	12	Second-Year Graduate Study	3		
			Third-Year Graduate Study	7		

**Table 2:** Mood scores of all participants after listening to cheerful music (*n* = 32) and melancholic music (*n* = 31).

Type of Music	*n*	Ordinal Mean	Mann–Whitney *U*	*p*
**Cheerful music**	32	33.59	445.000	0.394
**Melancholic** **music**	31	30.35		

**Table 3:** Descriptive statistics of Creative Feedback Perception Scale scores for professional and non-professional groups (*M* + *SD*).

Groups	Scale Score
**Design Professional Group**	7.639 ± 6.213
**Control Group**	5.212 ± 4.442

**Table 4:** Design of descriptive statistical tables (*M* + *SD*) of attention subnetworks in the professional group (*n* = 61) and control group (*n* = 33).

Attention Subnetwork	Groups	Cheerful Music	Melancholic Music	No Music
**Alerting**	Design Professional Group	35.509 ± 17.881	29.978 ± 19.863	20.387 ± 15.007
	Control Subjects	19.421 ± 23.625	30.062 ± 13.197	13.785 ± 12.209
**Orienting**	Design Professional Group	33.242 ± 22.865	48.22 ± 21.073	30.13 ± 18.406
	Control Subjects	29.546 ± 19.287	39.497 ± 21.988	38.074 ± 20.83
**Executive control**	Design Professional Group	77.534 ± 37.311	64.78 ± 34.641	63.853 ± 30.734
	Control Subjects	67.227 ± 35.026	63.388 ± 29.63	57.194 ± 26.02

**Table 5:** Statistics of ANOVA results for the three subsystems of the design professional group’s attentional network (alerting, orienting, and executive control).

Prerequisite	*N*	*F*	*p*	*η* ^2^
**Alerting**	61	3.812	0.028	0.116
**Orienting**	61	4.301	0.018	0.129
**Executive control**	61	1.021	0.367	0.034

## Data Availability

Data are included in the article.
